# Vanadium

**Published:** 1912-09

**Authors:** 


VANADIUM.—It is very unfortunate for the future of what promises apparently to be a valuable and unique therapeutic agent, the metal vanadium, that an article in the *Journal of the American Medical Association* for June 22, exposing an obesity "cure," should have involved a person who has become identified to a considerable extent with the manufacture of a line of proprietary remedies avowedly based upon the oxidizing properties of the curious metal. We hope to show shortly that vanadium, especially when combined with selenium, has remarkable properties hitherto totally unsuspected, and perchance may take a place in the professional armamentarium alongside its chemical brother, arsenic, and even share with mercury and quinine their indispensability to the prescriber. It is true that we may be too hopeful in our attitude toward this singular agent, but we ask of our readers an open mind, unprejudiced by anything they may have read concerning its exploitation, when we produce the preliminary evidence, along lines which no proprietary house has yet ventured to explore. What has been wanting so far is a standard solution or other preparation of vanadium which may be used by any pharmacist to prepare the extemporaneous prescription of the physician, and we have some reason to think that this will soon be ready. In the *Journal* for June 10, 1911, we published an editorial article on "The Possible Therapeutic Value of Vanadium," basing our conclusion upon certain reports we had read of its poisonous effects, and we are beginning to feel a little pride in that forecast.—*N. Y. Medical Journal.*

